# High-mobility and air-stable single-layer WS_2_ field-effect transistors sandwiched between chemical vapor deposition-grown hexagonal BN films

**DOI:** 10.1038/srep10699

**Published:** 2015-06-01

**Authors:** M Waqas Iqbal, M Zahir Iqbal, M Farooq Khan, M Arslan Shehzad, Yongho Seo, Jong Hyun Park, Chanyong Hwang, Jonghwa Eom

**Affiliations:** 1 Department of Physics and Graphene Research Institute, Sejong University, Seoul 143-747, Korea; 2 Faculty of Nanotechnology & Advanced Materials Engineering and Graphene Research Institute, Sejong University, Seoul 143-747, Korea; 3 Department of Materials Science and Engineering, Chungnam National University, Daejeon 305-764, Korea; 4 Center for Nanometrology, Korea Research Institute of Standards and Science, Daejeon 305-340, Korea

## Abstract

An emerging electronic material as one of transition metal dichalcogenides (TMDCs), tungsten disulfide (WS_2_) can be exfoliated as an atomically thin layer and can compensate for the drawback of graphene originating from a gapless band structure. A direct bandgap, which is obtainable in single-layer WS_2_, is an attractive characteristic for developing optoelectronic devices, as well as field-effect transistors. However, its relatively low mobility and electrical characteristics susceptible to environments remain obstacles for the use of device materials. Here, we demonstrate remarkable improvement in the electrical characteristics of single-layer WS_2_ field-effect transistor (SL-WS_2_ FET) using chemical vapor deposition (CVD)-grown hexagonal BN (h-BN). SL-WS_2_ FET sandwiched between CVD-grown h-BN films shows unprecedented high mobility of 214 cm^2^/Vs at room temperature. The mobility of a SL-WS_2_ FET has been found to be 486 cm^2^/Vs at 5 K. The ON/OFF ratio of output current is ~10^7^ at room temperature. Apart from an ideal substrate for WS_2_ FET, CVD-grown h-BN film also provides a protection layer against unwanted influence by gas environments. The h-BN/SL-WS_2_/h-BN sandwich structure offers a way to develop high-quality durable single-layer TMDCs electronic devices.

Despite all its advantages as an important material for atomically thin layered electronic device applications, graphene cannot be used as a promising material for active channel in field-effect transistors (FETs) because of the absence of a bandgap. Bandgap in graphene can be introduced by patterning into nanoribbons^1^, chemical functionalization^2^, and dual-gated bilayer graphene^3^, but always at the cost of significant mobility degradation. Moreover, their bandgap size is small, and the ON/OFF ratio is too small to be applicable to FETs. In contrary, several two-dimensional transition metal dichalcogenides (TMDCs) retain considerable bandgap around 1 eV to 2 eV^4^,^5^. Tungsten-based TMDCs compounds have shown a compelling thickness-dependent electronic band structure^6^,^7^ with relatively high carrier mobility^8^. As a tungsten based TMDCs compound, WS_2_ shows the transition of an indirect-to-direct bandgap when cleaved into monolayer^9^. Bulk WS_2_ is a semiconductor with an indirect bandgap of 1.4 eV, but monolayer WS_2_ presented a direct bandgap of 2.1 eV^10^. WS_2_ crystal is formed by layers of covalently bonded in-plane S-W-S atoms. These atoms compose two sheets of S and one sheet of W atoms that are hexagonally packed^11^. Adjacent layers in WS_2_ crystals are bound together by weak van der Waals forces. Given these weak interlayer interactions^12^,^13^, WS_2_ can be fabricated into single or a few layers by micromechanical cleavage method.

WS_2_ is currently a focus as next-generation nanoelectronic and optoelectronic materials. The material retains extremely high ON/OFF current ratio, high thermal stability, absence of dangling bonds, and electrostatic integrity^14^. Atomically thin layer of WS_2_ is becoming a new competitor to graphene, as well as traditional semiconductors, in a variety of applications, such as low power FETs, optoelectronic devices, memory devices, and chemical sensors. However, WS_2_ based devices suffer degradation of intrinsic properties and overall permanence because of environmental effects. In previous reports, single-layer (SL)-WS_2_ on Si/SiO_2_ substrate showed mobilities ranging between 40 and 60 cm^2^/Vs at room temperature^15^,^16^, because its electrical transport properties were strongly affected by interfacial charged impurities, surface roughness on Si/SiO_2_ substrates^17^,^18^. Suspending geometry^19^ may offer considerable improvements in intrinsic electrical properties of WS_2_. However, this kind of geometry imposes severe limitations on device fabrication. The improvement in sample quality in a substrate-supported geometry is necessary for the future progress of WS_2_ device technology. Efforts have been exerted to develop alternatives to the substrates. An ideal choice for alternative substrate is hexagonal BN (h-BN), which can be used to eliminate problematic surface effects in WS_2_ samples^20^, because h-BN has a large bandgap, is comparatively inert, does not possess dangling bonds, possesses low density of charged impurities, and is naturally flat^21^,^22^.

In this paper, we have developed high-mobility SL-WS_2_ FETs using chemical vapor deposition (CVD)-grown h-BN. Metal electrodes to SL-WS_2_ were constructed of Al and Au to achieve ohmic contact for improvement of device characteristics. The SL-WS_2_ FET sandwiched between CVD-grown h-BN films showed unprecedented mobilities of 185 cm^2^/Vs at room temperature and 486 cm^2^/Vs at 5 K. We also found that another SL-WS_2_ FET sandwiched between CVD-grown h-BN films showed the mobility of 214 cm^2^/Vs at room temperature. The ON/OFF ratio of output current is ~10^7^ at room temperature. Whereas hysteresis was found in transfer characteristics for WS_2_ FETs on Si/SiO_2_ substrate, this occurrence was absent for WS_2_ FETs sandwiched between CVD-grown h-BN films. The CVD-grown h-BN film provides a stable platform for WS_2_ FETs and works as a protection layer against external environments.

## Results and Discussion

### Characterization of single-layer WS_2_ by optical and atomic force microscopy

**Figure 1:**
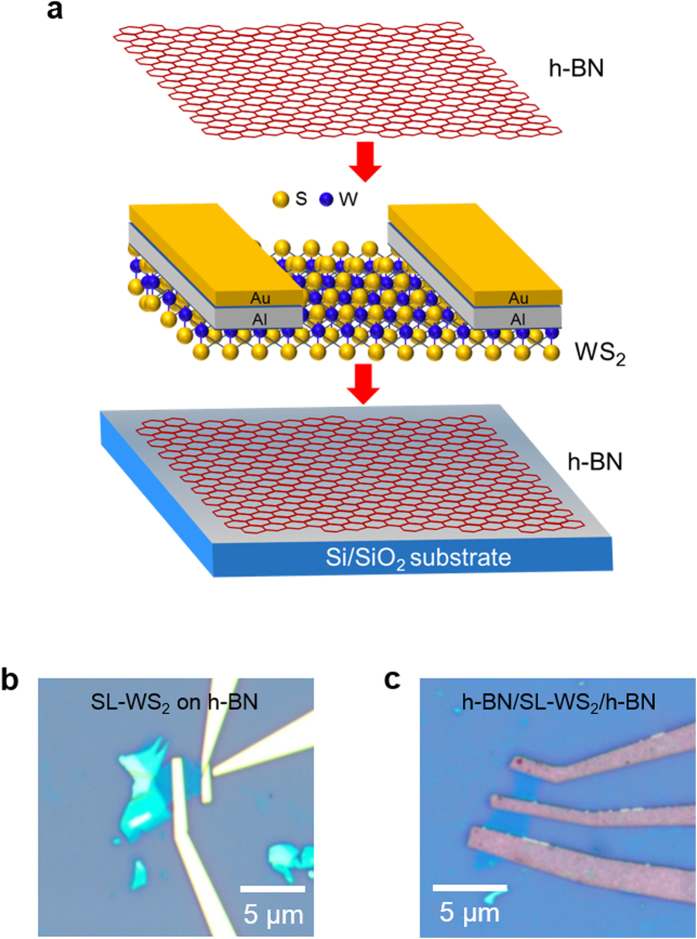
Optical image. (**a**) Schematic of a h-BN/SL-WS_2_/h-BN field-effect transistor. (**b**) Optical image of the mechanically exfoliated single-layer WS_2_ film on CVD-grown h-BN film. (**c**) Optical image of the mechanically exfoliated single-layer WS_2_ sandwiched between h-BN films (h-BN/SL-WS_2_/h-BN). The electrical contacts to WS_2_ films were made of Al/Au.

Figure 1a shows the schematic of a WS_2_ FET device sandwiched between CVD-grown h-BN films. The CVD-grown h-BN film was transferred on Si substrate with 300 nm thick SiO_2_ top layer, and then a SL-WS_2_ film was placed on top of the h-BN film by micromechanical cleavage method. The electrical contacts to the SL-WS_2_ film were constructed by e-beam lithography and thermal evaporation of Al (60 nm) and Au (40 nm) films, where the Au layer was deposited to prevent the deterioration of Al film. As a final cap layer, another CVD-grown h-BN film was transferred on top of the SL-WS_2_ device. Figure 1b shows the optical image of mechanically exfoliated SL-WS_2_ on CVD-grown h-BN with Al/Au contacts. Figure 1c shows the optical image of the mechanically exfoliated SL-WS_2_ device sandwiched between h-BN films.

**Figure 2:**
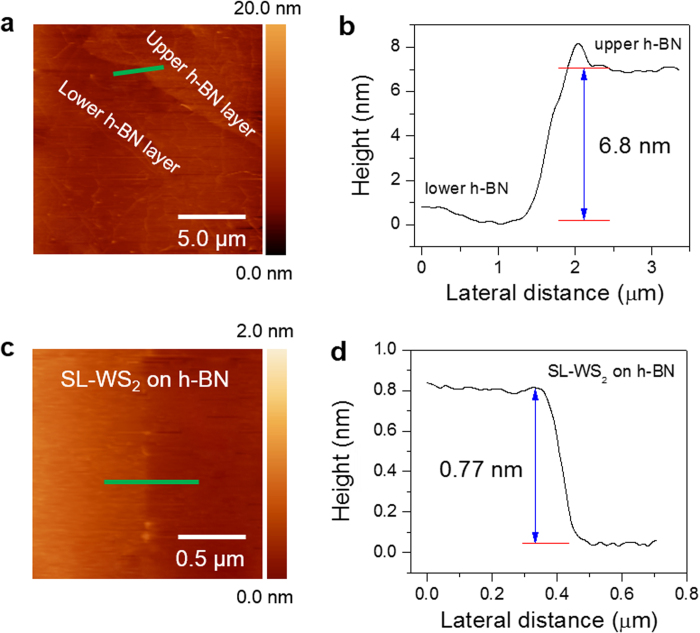
Atomic force microscopy. (**a**) Atomic force microscopy (AFM) of CVD-grown h-BN film on SiO_2_ substrate. (**b**) Thickness profile of CVD-grown h-BN film on SiO_2_ substrate along the green line in AFM image. The 6.8 nm thickness indicates nine layers of CVD-grown h-BN. (**c**) AFM image of single-layer WS_2_ flake on h-BN film. (**d**) Height profile of the single-layer WS_2_ along the green line in AFM image. The 0.77 nm thickness indicates one layer of WS_2_.

The thickness of CVD-grown h-BN and WS_2_ flakes were further verified by atomic force microscopy (AFM). The AFM image was obtained in tapping mode under ambient conditions. Figure 2a represents the surface topology and line profile of CVD-grown h-BN by AFM. In Fig. 2b, the thickness of the upper CVD-grown h-BN film is 6.8 nm, which corresponds to nine layers of h-BN. Given that the bottom h-BN film was also transferred from the same batch of CVD-grown h-BN, the number of layers should be same. Figure 2c shows the surface topology of the SL-WS_2_ film on h-BN obtained by AFM. The surface of WS_2_ film was uniform with extremely low roughness. In Fig. 2d, the thickness of the SL-WS_2_ film was measured as 0.77 nm on h-BN substrate.

### Transport properties of SL-WS_2_ FETs on SiO_2_ substrate with Al/Au contact

**Figure 3:**
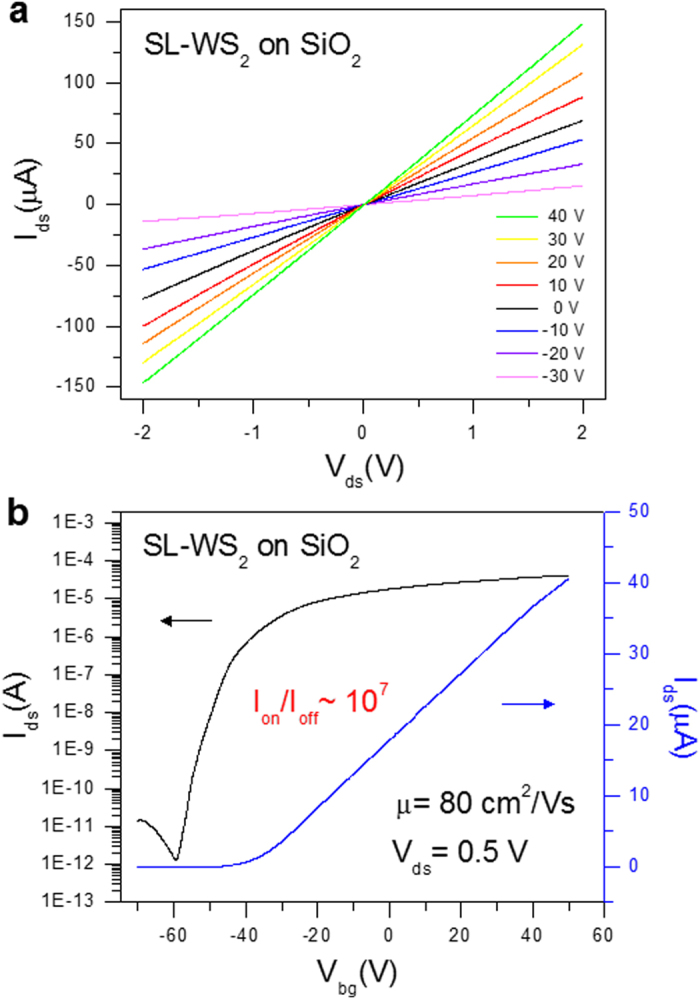
Transport properties of SL-WS_2_ FETs on SiO_2_ substrate. (**a**) Output characteristics (*I*_ds_–*V*_ds_) of SL-WS_2_ FET at different back-gate voltages ranging from –30 V to +40 V in steps of 10 V. (**b**) Transfer characteristics (*I*_ds_–*V*_bg_) of the SL-WS_2_ FET on SiO_2_ substrate with Al/Au contacts. ON/OFF ratio of the device is ~10^7^ at room temperature.

The electrical characteristics of the device were investigated at room temperature under vacuum. The electrical contacts to WS_2_ films also perform an important function in device performance. Recent studies showed that the electrical device performance of TMDC FETs can be critically influenced by contact resistances^23^, and the performance was conventionally limited by Schottky barriers at the metal/semiconductor interfaces^24^. One key factor in improving device performance involves the realization of ohmic contacts on WS_2_ films^25^. Prior to evaporation of contact metals in this experiment, we exposed WS_2_ films by deep ultraviolet light (with a dominant wavelength of λ = 220 nm and an average intensity of 11 mW/cm^2^) under a continuous N_2_ gas flow for 5 min to remove any oxygen or oxygen-derived group present at the WS_2_ surface^26^,^27^. After device fabrication, all devices were annealed in a tube furnace at a temperature of 200 °C under Ar/H_2_ gas flow for 4 h to remove the residues of e-beam or photolithography resists. Output characteristic curves (*I*_ds_–*V*_ds_) at various back-gate voltages ranging from –30 V to +40 V for the SL-WS_2_ FET are shown in Fig. 3a. The linear *I*_ds_–*V*_ds_ characteristic was obtained for the Al/Au (60/40 nm) contacts, whereas nonlinear *I*_ds_–*V*_ds_ characteristic was observed for Cr/Au (10/80 nm) contacts as shown in Figure S2b of supplementary information. The *I*_ds_–*V*_ds_ characteristics indicate that the Al/Au contact makes lower Schottky barrier height at the metal-to-WS_2_ interface in comparison with the case of Cr/Au contact. The lower Schottky barrier height is due to the work function of Al (~4.1 eV) being comparable to the electron affinity of WS_2_ film on SiO_2_. On the other hand the work function of Au (~5.1 eV) is much larger than the electron affinity of WS_2_ film, which yields to a relatively high Schottky barrier height.

Figure 3b represents the transfer characteristics (*I*_ds_–*V*_bg_) of SL-WS_2_ FET on SiO_2_ substrate at *V*_ds_ = 0.5 V. The black curve in the graph is plotted in the logarithmic scale for the *I*_ds_–*V*_bg_ curve. The output current ON/OFF ratio for the SL-WS_2_ FET is ~10^7^, and the threshold voltage (*V*_th_) was approximately –48 V, indicating n-type doping state. The threshold voltage is defined as the intercept of the *V*_bg_ axis obtained by extrapolating the linear portion of the curve of *I*_ds_–*V*_bg_ curve. The field-effect mobility (μ) of SL-WS_2_ FET is 80 cm^2^/Vs. Field-effect mobility was obtained by the equation
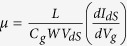
, where *L* is the channel length, *W* is the channel width, 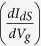
is the slope of transfer characteristic of the device at *V*_ds_ = 0.5 V, and *C*_g_ is the gate capacitance of ∼105 aF/μm^2^ for our Si/SiO_2_ substrate^22^.

### Transport properties of SL-WS_2_ FETs on CVD-grown h-BN films

**Figure 4:**
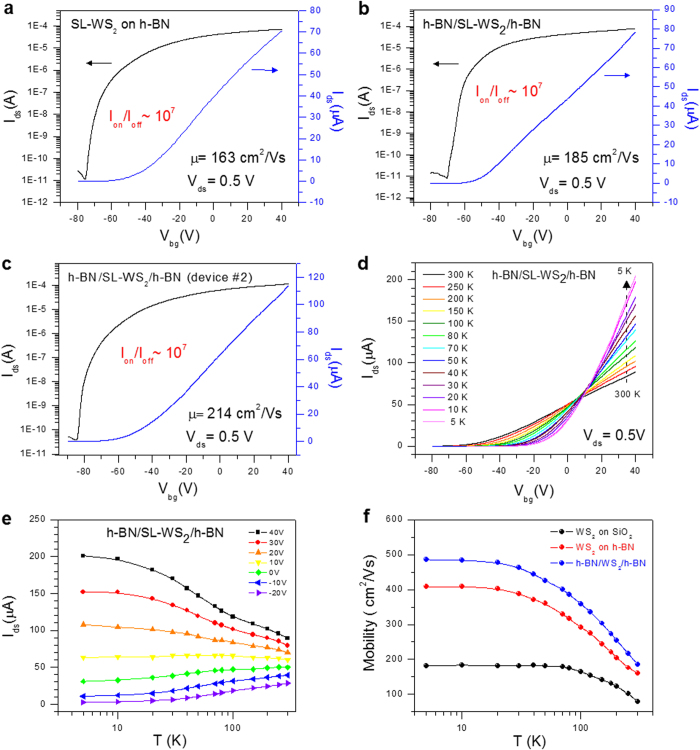
Transport properties of SL-WS_2_ FETs on CVD h-BN film. (**a**) Transfer characteristics (*I*_ds_–*V*_bg_) of the mechanically exfoliated SL-WS_2_ FET on CVD-grown h-BN film at 300 K. (**b**) Transfer characteristics (*I*_ds_–*V*_bg_) of the mechanically exfoliated SL-WS_2_ FET enclosed by h-BN at 300 K. ON/OFF ratio of the device is ~10^7^. (**c**) Transfer characteristics (*I*_ds_–*V*_bg_) of the mechanically exfoliated SL-WS_2_ FET enclosed by h-BN (device #2) at 300 K. (**d**) Transfer characteristics (*I*_ds_–*V*_bg_) of the mechanically exfoliated SL-WS_2_ FET enclosed by h-BN films at different temperatures. (**e**) Output current as function of temperature for different values of the back-gate voltage. (**f**) Electron field-effect mobility of SL-WS_2_ FETs on different substrates at various temperatures.

Figure 4a represents the transfer characteristics (*I*_ds_–*V*_bg_) of SL-WS_2_ FET on CVD-grown h-BN film at *V*_ds_ = 0.5 V, where the top h-BN film was absent. Field-effect mobility was 163 cm^2^/Vs at room temperature. The output current ON/OFF ratio for SL-WS_2_ FET on CVD-grown h-BN film is ~10^7^, and *V*_th_ approximated –58 V. Notably, both μ and ON/OFF ratio were improved by changing the substrate from SiO_2_ to h-BN film. The characteristics of SL-WS_2_ FET can be further improved by adding a top layer of h-BN film. Figure 4b represents the transfer characteristics (*I*_ds_–*V*_bg_) of SL-WS_2_ FET sandwiched between CVD-grown h-BN films at *V*_ds_ = 0.5 V. The output current ON/OFF ratio of the device is ~10^7^, and μ was 185 cm^2^/Vs. Similar results were reproducibly obtained for other h-BN/SL-WS_2_/h-BN devices, as shown in Fig. 4c (See also Figure S3 of supplementary information). We demonstrated the mobility of 214 cm^2^/Vs at room temperature for the h-BN/SL-WS_2_/h-BN (device #2).

### Temperature-dependent electrical transport properties of SL-WS_2_ FETs

We have investigated the temperature-dependent electronic transport properties of SL-WS_2_ FETs. Figure 4d shows the transfer characteristics (*I*_ds_–*V*_bg_) of the SL-WS_2_ FET sandwiched between CVD-grown h-BN films at *V*_ds_ = 0.5 V at different temperatures. For V_bg_ < 10 V the SL-WS_2_ FET behaves as a traditional semiconductor with conductance decreasing as the temperature is decreased. In comparison, for V_bg_ ⊠ 10 V, conductance increases as temperature is decreased. Semiconductor-to-metal transition was observed when *V*_bg_ is increased to 10 V. This result suggests that a degenerately doped state is realized in SL-WS_2_ film for V_bg_ > 10 V. Figure 4e shows the temperature dependence of *I*_ds_ for different values of *V*_bg_. Here, we can clearly see the critical *V*_bg_ of 10 V, at which *I*_ds_ remains almost independent of temperature. However, the *I*_ds_ of SL-WS_2_ FET increases with decreasing temperature for V_bg_ > 10 V, indicating metallic behavior. For V_bg_ < 10 V, *I*_ds_ decreases with decreasing temperature, indicating a semiconducting behavior. Observations of a similar semiconductor-to-metal transition were reported in other TMDCs materials^28^,^29^.

We have further investigated the electron filed effect mobility of SL-WS_2_ FET on different substrates at various temperatures. The temperature dependence of μ of SL-WS_2_ FETs is compared in Fig. 4f. The electron field-effect motilities of SL-WS_2_ FETs on SiO_2_, h-BN, and h-BN/SL-WS_2_/h-BN were 80, 163, and 185 cm^2^/Vs, respectively, at T = 300 K, and 180, 408, and 486 cm^2^/Vs, respectively, at T = 5 K. For the entire temperature range in this experiment, the h-BN/SL-WS_2_/h-BN device showed the highest mobility. The electron field-effect mobility of SL-WS_2_ on SiO_2_ substrate starts to saturate below 70 K, but that of SL-WS_2_ on h-BN films is saturated below 20 K. This result suggests that scattering factors influencing electron transport in the SL-WS_2_ film can be significantly reduced using h-BN films as substrate.

Among the scattering factors, charge impurities in substrate may dominantly influence the electron transport in SL-WS_2_ film. One of advantages for h-BN substrate includes its capability to provide charge impurity-free environment. To verify the role of our CVD-grown h-BN films, we investigated the existence of hysteresis in the transfer characteristics of SL-WS_2_ FETs by sweeping V_bg_^18^. Figure S5a in supplementary information shows a hysteresis curve, which is typically observed in SL-WS_2_ FET on SiO_2_ substrate. However, transfer characteristics of SL-WS_2_ FET sandwiched between CVD-grown h-BN (h-BN/SL-WS_2_/h-BN) shows virtually no hysteresis (Figure S5b in supplementary information). The hysteresis indicates that a number of charge impurities exist in the SiO_2_ substrate, whereas extremely few charge impurities are present in CVD-grown h-BN.

### Raman spectra of SL-WS_2_ FETs on different substrates

**Figure 5:**
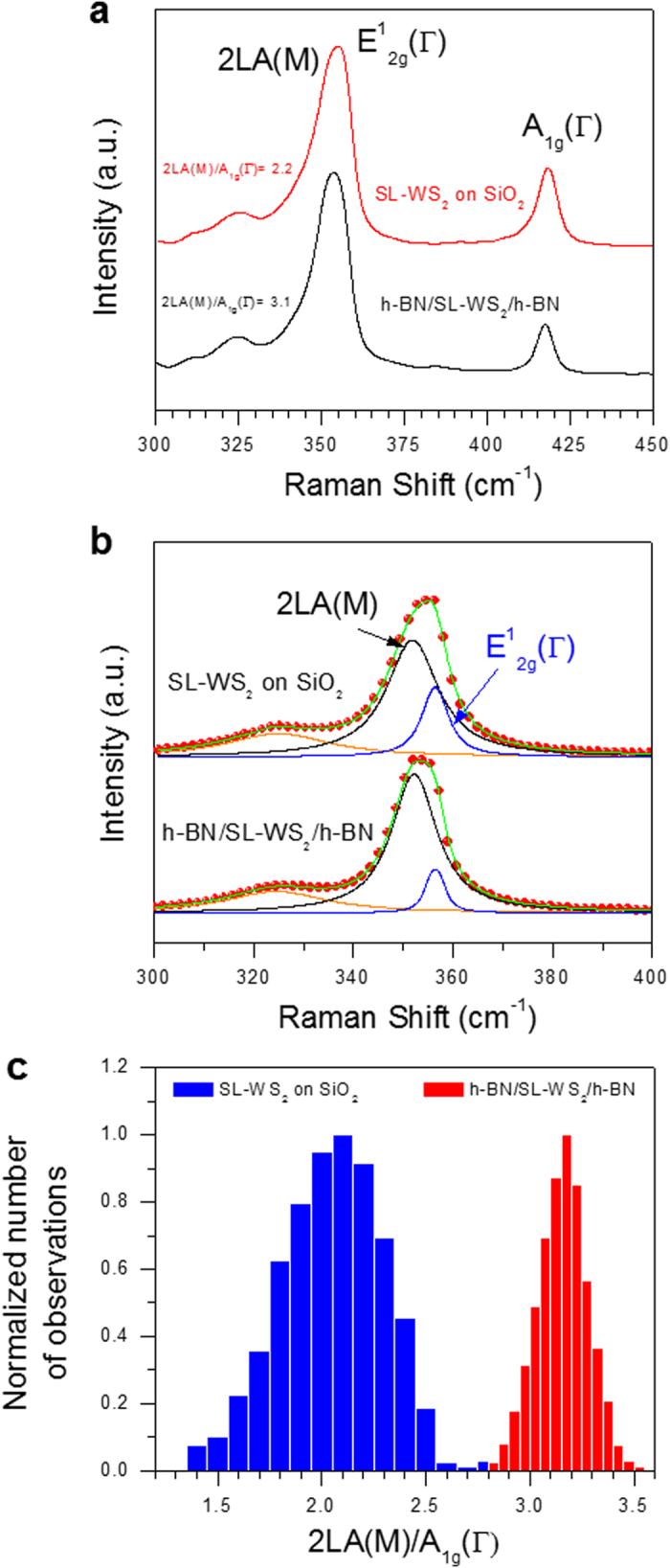
Raman spectra of WS_2_ films on different substrates. (**a**) Raman spectra for SL-WS_2_ on SiO_2_ and h-BN/SL-WS_2_/h-BN. (**b**) Lorentzian fitting for E_2g_ and 2LA(M) peaks. Red circles represent experimental data, while blue, black, and green lines represent E_2g_, 2LA(M), and combined peak fitting, respectively. (**c**) Statistical distribution of the Raman intensity ratio (I_2LA(M)_/I_A1g_) for SL-WS_2_ on SiO_2_ substrate, h-BN/SL-WS_2_/h-BN. The mean value of I_2LA(M)_/I_A1g_ was 2.0 for SL-WS_2_ on SiO_2_ and 3.1 for h-BN/SL-WS_2_/h-BN.

Structural characterizations of SL-WS_2_ films in the devices on different substrates (SiO_2_ and h-BN) were performed by Raman spectroscopy. Figure 5a shows a Raman shift for SL-WS_2_ film on SiO_2_ and h-BN/SL-WS_2_/h-BN. The Raman spectra of SL-WS_2_ films exhibited strong signals of in-plane E^1^_2g_, out-of-plane A_1g_, and vibration second-order 2LA(M) modes^9^. The first-order E^1^_2g_ and A_1g_ optical modes were considered to explain the properties of two-dimensional material, such as MoS_2_ in the previous report^30^. However, the intensity of the 2LA(M) mode at 352 cm^–1^ was distinctly predominant for WS_2_. Although the 2LA(M) mode overlapped with the first-order E^1^_2g_ mode at 355.4 cm^–1^, multi-peak Lorentzian fitting can clarify their individual contributions as seen in Fig. 5b^10^. The Raman peak positions of E^1^_2g_ and A_1g_ for SL-WS_2_ are 355.4 and 417.7 cm^–1^, respectively. The frequency difference between Raman A_1g_ and E^1^_2g_ modes (Δ = A_1g_ – E^1^_2g_) is about 62.3 cm^–1^, which indicates a single-layer WS_2_ film. The wave number difference between 2LA(M) and A_1g_ modes can also be used to identify the layer number of a WS_2_ film^9^,^10^,^13^. The wave number differences between 2LA(M) and A_1g_ modes are 65.3 cm^–1^ for SL-WS_2_ films in the devices. Figures 5c shows statistical distribution of the Raman intensity ratio (I_2LA(M)_/I_A1g_) for SL-WS_2_ on SiO_2_ substrate and h-BN/SL-WS_2_/h-BN. Statistical distribution was taken for the area of 5 × 5 μm^2^ in SL-WS_2_ on different substrates. The normalized number of observations shows the distribution of Raman shift observations in the scanned area. The most probable ratio of I_2LA(M)_/I_A1g_ was 2.0 for SL-WS_2_ on SiO_2_ and 3.1 for h-BN/SL-WS_2_/h-BN. Figure 5c also indicates the homogeneity of SL-WS_2_ quality on different substrates. Larger standard deviation of intensity ratio of I_2LA(M)_/I_A1g_ was found for SL-WS_2_ on the SiO_2_ substrate, whereas smaller standard deviation was found for h-BN/SL-WS_2_/h-BN. This finding indicates that a higher uniformity of SL-WS_2_ quality can be achieved by enclosing the SL-WS_2_ with h-BN films.

## Conclusion

In summary, a SL-WS_2_ FET of unprecedented high quality has been achieved by CVD-grown h-BN films as substrate and capping layer. Electrical transport measurements revealed that SL-WS_2_ FET on h-BN film exhibited high-mobility and transfer characteristics that are free of charged impurities in comparison with SL-WS_2_ FET on SiO_2_. The field-effect mobility of h-BN/SL-WS_2_/h-BN was 185 cm^2^/Vs at 300 K and 486 cm^2^/Vs at 5 K. The highest mobility was found to be 214 cm^2^/Vs for a h-BN/SL-WS_2_/h-BN device at room temperature. Semiconductor-to-metal transition was also observed when *V*_bg_ was increased over 10 V. Apart from providing an ideal substrate for WS_2_, CVD-grown h-BN film also imparted a protection layer preventing unwanted environmental effects. The h-BN/SL-WS_2_/h-BN structure offered considerable advantages in fabricating stable WS_2_ electronic devices. This work has demonstrated the potential application of large-area growth of h-BN and the simplified fabrication of h-BN/SL-WS_2_/h-BN devices to enhance transport characteristics.

## Experimental section

### Transfer method

For the transfer of CVD-grown h-BN film, polymethyl methacrylate (PMMA) was spin-coated on CVD-grown h-BN film on Cu foil. Next, the Cu foil was etched out by soaking in an ammonium persulfate solution for 24 h. Finally, the h-BN/PMMA film was transferred onto a Si substrate. After the PMMA film was removed by soaking in acetone, CVD-grown h-BN film on the Si substrate with a 300 nm-thick SiO_2_ capping layer was obtained. Then, the CVD-grown h-BN film on SiO_2_ substrate was placed in an oxygen plasma etching system to remove the remaining PMMA residue for 2 min. Exfoliated single-layer WS_2_ films were obtained from natural bulk crystals of WS_2_ by subsequent transfer of the h-BN films on 300 nm-thick SiO_2_ substrate using standard Scotch tape method. Structural morphology, thickness, and topography of single-layer WS_2_ films were examined using optical microscopy, Raman spectroscopy, and AFM, respectively. The laser wavelength of the Raman micro-spectrometer was 514 nm, and the power was maintained at below 1.0 mW to prevent laser-induced heating. The laser spot size of Raman spectroscopy was 0.7 μm for the wavelength of 514 nm.

### Device Fabrication and Characterizations

We fabricated single-layer WS_2_ devices by photolithography, e-beam lithography, and O_2_ plasma etching. Large electrode patterns with Cr/Au (6/30 nm) film were deposited using a thermal evaporator after standard photolithography. E-beam lithography was then employed to pattern source and drain contacts, from which the film was made by evaporation of Al/Au (60/40 nm). Prior to the evaporation of contact metals, we exposed the WS_2_ films by deep ultraviolet light with a dominant wavelength of λ = 220 nm and an average intensity of 11 mW/cm^2^ in a continuous N_2_ gas flow for 5 min. This process aimed to remove any oxygen or oxygen-derived group present at the WS_2_ surface. After device fabrication, all devices were annealed in a tube furnace at a temperature of 200 °C under Ar/H_2_ (97.5% Ar/2.5% H_2_) gas flow for 4 h. Electrical transport measurements were carried out at room temperature under vacuum.

### Synthesis of h-BN

The growth of h-BN film was performed on 25-μm-thick Cu foil (Alfa Aesar, 99.8% pure) using thermal CVD. To remove the impurities and obtain the flatness of the Cu foil, we applied a mechanical polishing process followed by a short electro-polishing. The Cu foil was annealed at 990 °C for 30 min with H_2_ gas at a flow rate of 5 standard cubic centimeters per minute (sccm) to remove the oxide layer. Ammonia borane (Sigma-Aldrich, 97% pure) was thermally decomposed to hydrogen, aminoborane, and borazine at a temperature range from 80 to 120 °C. After the thermal cleaning, h-BN was synthesized with borazine gas and hydrogen at 997 °C for 30 min. The furnace was cooled from 997 to 500 °C at a rate of ~35 °C/min after the synthesis of h-BN films.

## Additional Information

**How to cite this article**: Iqbal, M. W. *et al.* High-mobility and air-stable single-layer WS_2_ field-effect transistors sandwiched between chemical vapor deposition-grown hexagonal BN films. *Sci. Rep.* **5**, 10699; doi: 10.1038/srep10699 (2015).

## Supplementary Material

Supplementary Information
