# Supplementary material for: High-mobility and air-stable single-layer WS2 field-effect transistors sandwiched between chemical vapor deposition-grown hexagonal BN films
Source: Sci Rep. 2015 Jun 1;5:10699. doi: 10.1038/srep10699 (PMC4450543; doi:10.1038/srep10699)
Supplement: Supplementary Information [file srep10699-s1.doc]

**Supplementary Information**

**High-mobility and air-stable single-layer WS_2_ field-effect transistors sandwiched between chemical vapor deposition-grown hexagonal BN films**

**M Waqas Iqbal^1^, M Zahir Iqbal^1^, M Farooq Khan^1^, M Arslan Shehzad^2^, Yongho Seo^2^, Jong Hyun Park^3^, Chanyong Hwang^4^, Jonghwa Eom^1*^**

^1^Department of Physics and Graphene Research Institute, Sejong University, Seoul 143-747, Korea

^2^Faculty of Nanotechnology & Advanced Materials Engineering and Graphene Research Institute, Sejong University, Seoul 143-747, Korea

^3^Department of Materials Science and Engineering, Chungnam National University, Daejeon 305-764, Korea

^4^Center for Nanometrology, Korea Research Institute of Standards and Science, Daejeon 305-340, Korea

*E-mail: [eom@sejong.ac.kr](mailto:eom@sejong.ac.kr)

The h-BN film was grown on Cu foils by thermal chemical vapor deposition (CVD) as shown schematically in Figure S1a. Ammonia borane (Sigma-Aldrich, 97% pure) was used as a precursor to make h-BN films. It was thermally decomposed into hydrogen, aminoborane, and borazine at a temperature range from 80 to 120 °C, and then aminoborane was trapped by the filter. The growth of h-BN film was performed on 25-μm-thick Cu foil (Alfa Aesar, 99.8% pure). The mechanically and electro-polished Cu foil was annealed at 990 °C for 30 min with H_2_ gas at a flow rate of 5 standard cubic centimeters per minute. After the cleaning process, h-BN films were synthesized with borazine gas and hydrogen at 997 °C for 30 min. After the synthesis of h-BN films, the furnace was cooled from 997 to 500 °C at a rate of ~35 °C/min.

Optical micrograph images of CVD-grown h-BN used in our study is shown in Figure S1b. Raman spectroscopy of CVD-grown h-BN transferred on Si/SiO_2_ substrate is shown in Figure S1c[^1^](#_ENREF_1), and the h-BN peak was observed at 1369 cm^–1^, which confirmed the stable growth and clean transfer of the h-BN film from Cu foil. We used atomic force microscopy (AFM) to confirm the thickness and morphology of CVD-grown h-BN films.

We have fabricated SL-WS_2_ FETs on SiO2 substrates, of which optical microscope images are shown in Figure S2. The SL-WS_2_ FET with Cr/Au (10 nm/80 nm) contacts is shown in Figure S2a, whereas the SL-WS_2_ FET with Al/Au (60 nm/40 nm) contacts is shown in Figure S2b. To check the electrical characteristics of the device, electrical transport measurement was performed at room temperature under vacuum. Figure S3a shows the transfer characteristics (*I*_ds_–*V*_bg_) of the single-layer WS_2_ (SL-WS_2_) field-effect transistor (FET) on SiO_2_ substrate with Cr/Au contact at a fixed source-drain voltage, *V*_ds_ = 0.5 V, after exposing the device to deep ultraviolet light in a continuous N_2_ gas flow (DUV + N_2_) for 30 min. The DUV light presents a dominant wavelength of *λ* = 220 nm and an average intensity of 11 mW/cm^2^. The black curve in the graph is plotted in the logarithmic scale for the *I*_ds_–*V*_bg_ curve. The ON/OFF ratio of the device is ~10^6^. The field-effect mobility of SL-WS_2_ FET after 30 min DUV + N_2_ treatment is 17 cm^2^/Vs. Output (*I*_ds_–*V*_ds_) characteristic curves at various gate voltages from –30 V to +40 V in steps of 10 V for SL-WS_2_ FET are shown in Figure S3b. Nonlinear *I*_ds_–*V*_ds_ characteristics suggest the existence of Schottky barriers between Cr/Au contact and WS_2_ film.

We fabricated several SL-WS_2_ FETs sandwiched between h-BN films (h-BN/SL-WS_2_/h-BN) with Al/Au (60 nm/40 nm) contacts to check the consistency of superior characteristics. Figure S4a represents the transfer characteristics (*I_ds_*-*V_bg_*) of one of h-BN/SL-WS_2_/h-BN devices. The ON/OFF ratio of the device is ~10^7^. The field-effect mobility of SL-WS_2_ FET was 214 cm^2^/Vs at room temperature. Figure S4b represents output characteristics (*I*_ds_–*V*_ds_) of h-BN/SL-WS_2_/h-BN at different back-gate voltages ranging from –30 V to +40 V in the steps of 10 V. The linear *I*_ds_–*V*_ds_ characteristics suggest ohmic contact between Al/Au and WS_2_ film.

We checked the role of top h-BN film as protection layer against oxygen environments. Figure S4c represents the transfer characteristics (*I*_ds_–*V*_bg_) of the h-BN/SL-WS_2_/h-BN device after exposure to DUV light in a continuous O_2_ gas flow (DUV + O_2_) for a certain time. A slight change was observed in the transfer characteristics of h-BN/SL-WS_2_/h-BN after 30 min DUV + O_2_ treatment. However, the change of threshold voltage (*V*_th_) of h-BN/graphene/h-BN was small, and the shift of *V*_th_ was only 4 V after 30 min DUV + O_2_ treatment. The h-BN/SL-WS_2_/h-BN sandwich structure offers an advantage for manufacturing stable WS_2_ electronic devices. The van der Waals interaction between WS_2_ and h-BN may obstruct O_2_ molecules from penetrating into the interface between WS_2_ and h-BN. The advantages of h-BN as a substrate for 2-dimensional materials were examined in previous reports.

The effect of DUV illumination in an N_2_ gas environment (DUV + N_2_) involved n-type doping[^4^](#_ENREF_4). The n-type doping effect for WS_2_ FETs by DUV + N_2_ treatment can be considered as the removal of O_2_ molecules from WS_2_ surface. The oxygen atoms/molecules on the surface of WS_2_ film may work as acceptors to draw electrons in the WS_2_ layer. Therefore, the removal of oxygen significantly increases drain-to-source current in the WS_2_ film. Figure S5a represents the transfer characteristics (*I*_ds_–*V*_bg_) of SL-WS_2_ FET on SiO_2_ substrate with Cr/Au contact before and after 30 min DUV + N_2_ treatment. The electron field effect mobility was 4 cm^2^/Vs prior to DUV + N_2_ treatment, and then increased to 17 cm^2^/Vs after DUV + N_2_ treatment. Figure S5b represents the transfer characteristics (*I*_ds_–*V*_bg_) of SL-WS_2_ FET on SiO_2_ substrate with Al/Au contact before and after 30 min DUV + N_2_ treatment. The electron field effect mobility was measured as 24 cm^2^/Vs before DUV + N_2_ treatment, becoming 80 cm^2^/Vs after 30 min DUV + N_2_ treatment. All measurements were performed in vacuum at T = 300 K. For comparison, the detail discussion on exposure of WS_2_ devices without top h-BN layer to DUV under gas environments can be found in our recent paper[^4^](#_ENREF_4).

To verify the role of our CVD-grown h-BN films, we investigated the existence of hysteresis in the transfer characteristics of SL-WS_2_ FETs by sweeping *V*_bg_. Figure S6a in supporting information shows a hysteresis curve, which is typically observed in SL-WS_2_ FET on SiO_2_ substrate, and where *V*_bg_ was swept continuously from −70 to +40 V and from +40 V to −70 V. We note that *V*_th_ moved toward negative (positive) values when *V*_bg_ was swept from −70 V (+40 V) to +40 V (−70 V). The hysteresis in *V*_th_ is 13 V for SL-WS_2_ FET on SiO_2_ substrate, which is due to charge impurities in SiO_2_ substrate[^5^](#_ENREF_5),[^6^](#_ENREF_6). Similar transfer characteristics were investigated for SL-WS_2_ FET sandwiched between CVD-grown h-BN (h-BN/SL-WS_2_/h-BN) as shown in Figure S6b. Virtually no hysteresis in *V*_th_ was observed for h-BN/SL-WS_2_/h-BN. The hysteresis indicates that a number of charge impurities exist in the SiO_2_ substrate, whereas extremely few charge impurities are present in CVD-grown h-BN.

We have measured the contact resistance of SL-WS_2_ by using transfer length method (TLM) for different metal contacts (Cr/Au, Al/Au). Figure 7a represents the optical image of SL-WS_2_ device with Cr/Au (10/80 nm) and Al/Au (60/40 nm) contacts to measure the contact resistance (*R*_c_). Since resistance (*R*) depends on the sample channel width (*W*), the specific resistance (ρ = *RW*) normalized to the sample width was measured. Figure 7b shows the specific resistance as a function of distance between the contacts. The specific contact resistance (ρ_c_ = *R*_c_*W*) of the SL-WS_2_ device with Al/Au contact was 1.25 kΩµm, whereas it was 6.55 kΩµm for Cr/Au contact. It is clear that the contact resistant is remarkably reduced by using Al/Au film, which leads to increase the drain-source current and enhances the device performance.


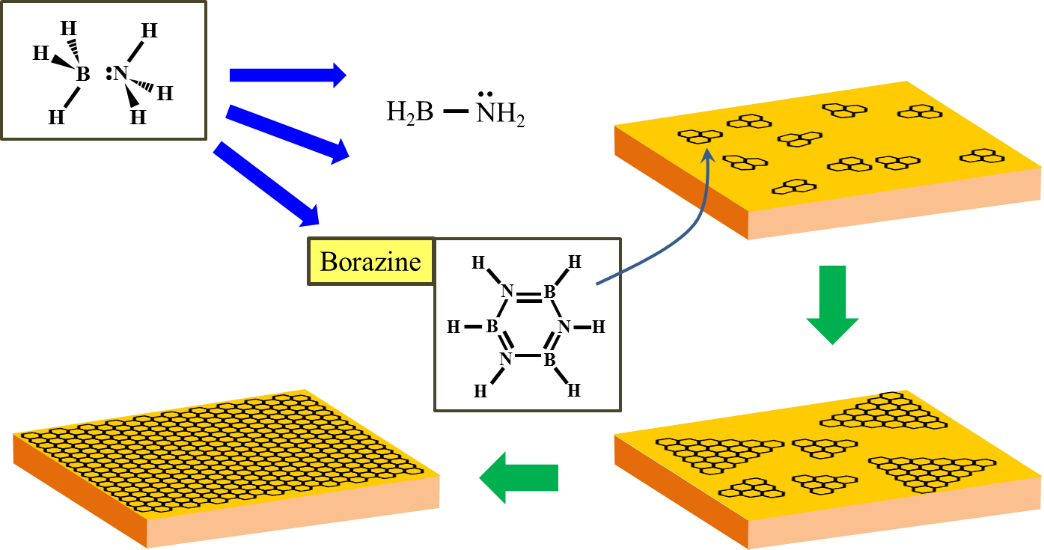


**a**

Ammonia borane

Hydrogen

Aminoborane

Thermal decomposition

(60°C ~ 180°C )

**b**


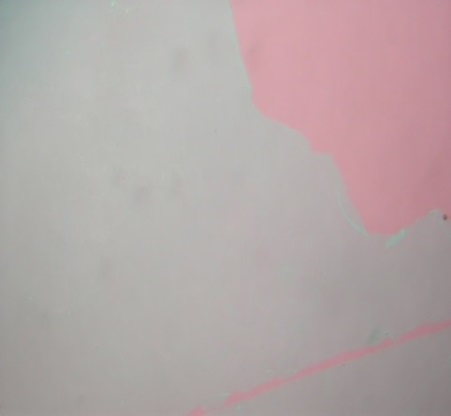


10 µm

h-BN

**Figure S1.** **Growth of h-BN by chemical vapor deposition method.** (**a**) Schematic of the h-BN growth process by thermal chemical vapor deposition (CVD). (**b**) Optical image of the CVD-grown h-BN after transfer onto Si/SiO_2_ substrate. (**c**) Raman spectra of CVD-grown h-BN transferred onto Si/SiO_2_ substrate. The h-BN peak was observed at 1369 cm^–1^, confirming the stable growth and clean transfer of h-BN onto Si/SiO_2_ substrate from Cu foil.


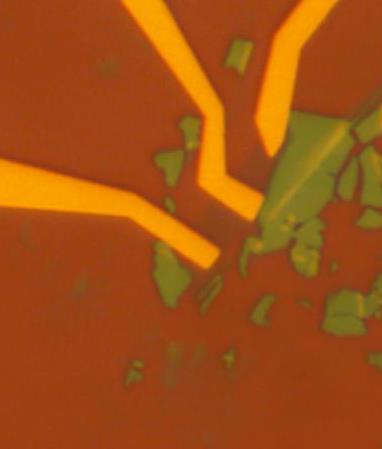


5µm

**a**

SL-WS_2_/SiO_2_


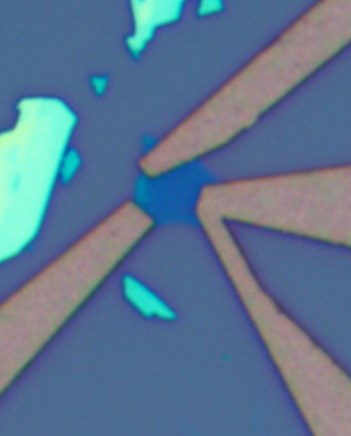


5µm

**b**

SL-WS_2_/SiO_2_

**Figure S2.** (**a**) SL-WS_2_ on SiO_2_ with Cr/Au contacts. (**b**) SL-WS_2_ on SiO_2_ with Al/Au contacts.

**Figure S3.** **Transport characteristics of SL-WS_2_ FET on SiO_2_ Substrate.** (**a**) Transfer characteristics (*I*_ds_–*V*_bg_) of SL-WS_2_ FET on SiO_2_ substrate with Cr/Au contact. The ON/OFF ratio of the device is ~10^6^. (**b**) Output characteristics (*I*_ds_–*V*_ds_) of SL-WS_2_ FET at different back-gate voltages ranging from –30 V to +40 V in steps of 10 V.

**Figure S4. Transport properties of h-BN/SL-WS_2_/h-BN device.** (**a**) Transfer characteristics (*I*_ds_–*V*_bg_) of mechanically exfoliated SL-WS_2_ FET sandwiched between h-BN films with Al/Au contact. The ON/OFF ratio of the device is ~10^7^. Mobility of device is 214 cm^2^/Vs at room temperature. (**b**) Output characteristics (*I*_ds_–*V*_ds_) of h-BN/SL-WS_2_/h-BN at different back-gate voltages ranging from –30 V to +40 V in steps of 10 V. (**c**) Transfer characteristics (*I*_ds_–*V*_bg_) of h-BN/SL-WS_2_/h-BN after exposure to DUV + O_2_ treatment for a certain time. Since DUV + O_2_ treatments were applied right after DUV + N_2_ (30 min) treatment, the black curve represents the data before DUV+O_2_ exposure.

**Figure S5. Transport properties of SL-WS_2_ FET on SiO_2_ substrate with different contact materials.** (**a**) Transfer characteristics (*I*_dS_–*V*_bg_) of SL-WS_2_ FET on SiO_2_ substrate with Cr/Au contact before and after 30 min DUV + N_2_ treatment. (**b**) Transfer characteristics (*I*_dS_–*V*_bg_) of SL-WS_2_ FET on SiO_2_ substrate with Al contact before and after 30 min DUV + N_2_ treatment. All measurements were performed in vacuum at T = 300 K.

**Figure S6. Hysteresis in transfer characteristics of SL-WS_2_ FETs on different substrates.** (**a**) Transfer characteristics (*I*_ds_–*V*_bg_) of SL-WS_2_ FET on SiO_2_ substrate, in which the back-gate voltage was swept continuously from −70 V to +40 V and from +40 V to −70 V. (**b**) Transfer characteristics (*I*_ds_–*V*_bg_) of the mechanically exfoliated SL-WS_2_ FET enclosed by h-BN films. Measurement was performed under vacuum at room temperature.


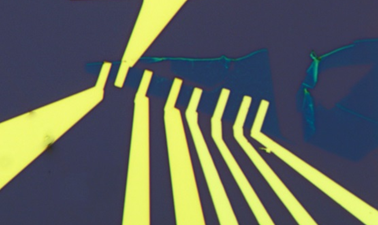


10µm

Cr/Au

Al/Au

SL-WS_2_

**a**

**Figure S7.** **Contact resistance measurement by transfer length method.** (**a**) Optical image of SL-WS_2_ device with Cr/Au (10/80 nm) and Al/Au (60/40 nm) contacts to measure the contact resistance by transfer length method. (**b**) Specific contact resistant (ρ_c_ = *R*_c_*W*) for the SL-WS_2_ device with Al/Au contact is 1.25 kΩμm, whereas it is 6.55 kΩµm for Cr/Au contact.

**References**

1. Iqbal MW, Iqbal MZ, Jin X, Eom J, Hwang C. Superior characteristics of graphene field effect transistor enclosed by chemical-vapor-deposition-grown hexagonal boron nitride. *J Mater Chem C* **2**, 7776-7784 (2014).

2. Levendorf MP*, et al.* Graphene and boron nitride lateral heterostructures for atomically thin circuitry. *Nature* **488**, 627-632 (2012).

3. Dean C*, et al.* Boron nitride substrates for high-quality graphene electronics. *Nature Nanotech* **5**, 722-726 (2010).

4. Iqbal MW, *et al.* Deep-ultraviolet-light-driven reversible doping of WS2 field-effect transistors. *Nanoscale* **7**, 747-757 (2015).

5. Late DJ, Liu B, Matte HR, Dravid VP, Rao C. Hysteresis in single-layer MoS2 field effect transistors. *Acs Nano* **6**, 5635-5641 (2012).

6. Joshi P, Romero H, Neal A, Toutam V, Tadigadapa S. Intrinsic doping and gate hysteresis in graphene field effect devices fabricated on SiO2 substrates. *J Phys Condens Matter* **22**, 334214 (2010).
